# Synthesis and Characterization of Fe^10^BO_3_/Fe_3_O_4_/SiO_2_ and GdFeO_3_/Fe_3_O_4_/SiO_2_: Nanocomposites of Biofunctional Materials

**DOI:** 10.1002/open.201300007

**Published:** 2013-05-13

**Authors:** Shanmin Gao, Xin Liu, Tao Xu, Xuehua Ma, Zheyu Shen, Aiguo Wu, Yinghuai Zhu, Narayan S Hosmane

**Affiliations:** [a] Department of Chemistry and Biochemistry, Northern Illinois University1425 W. Lincoln Hwy, DeKalb, IL 60115-2862 (USA); [b] School of Chemistry and Materials Science, Ludong UniversityYantai, 264025 (P. R. China); [c] Division of Functional Materials and Nano-devices, Ningbo Institute of Materials Technology & Engineering, Chinese Academy of SciencesNingbo, 315201 (P. R. China); [d] Institute of Chemical and Engineering Sciences (ICES)1 Pesek Road, Jurong Island, Singapore 627833 (Singapore)

**Keywords:** biofunctional materials, gel combustion techniques, nanocomposites, neutron capture therapy

Cancer is one of the leading causes of death worldwide; with many different types, it kills thousands of people every day. Various types of treatments have been developed to treat cancer, and new approaches that are currently under investigation include boron neutron capture therapy (BNCT)^1^ and gadolinium neutron capture therapy (GdNCT).^2^ Neutron capture therapy is primarily used to treat brain tumors, such as glioblastoma, a particularly aggressive type of brain tumor that is difficult to treat by conventional means such as radiation therapy. BNCT and GdNCT involve a bimodal approach to treatment, utilizing a cancer-specific drug and a neutron source (neutron beam). The approach is based on the ability of a boron isotope (^10^B) to absorb neutrons and emit localized cell-killing particles. The main mechanism that takes place in BNCT is the absorption of a neutron to convert ^10^B to ^11^B, with the release of ^4^He^2+^, ^7^Li^3+^, and energy.^3^ The energy that is released can then destroy the tumor cell. Gadolinium also attracted interest for its potential use in neutron capture therapy because it is the element with the highest cross-sectional value for thermal neutrons—2.55×10^5^ b and 6.10×10^4^ b for ^157^Gd and ^155^Gd, respectively.^4^ In fact, the thermal neutron value of ^157^Gd (2.55×10^5^ b) is 65 times that of ^10^B, and it releases Auger electrons, internal conversion electrons, γ-ray and X-ray after the capture of a single thermal neutron.[1, 5–7]

Targeted delivery of an anticancer drug is very desirable, as most of the commonly used agents have serious side effects associated with their use due to undesirable interactions with healthy cells. Moreover, targeted delivery can potentially enhance the therapeutic efficacy.^8^ Research on nanomaterials has grown explosively in the last few years, including an increased emphasis on developing nanomaterials as drug delivery vehicles.^9^, ^10^ The size of such delivery vehicles (<1000 nm) has attracted wide interest in the field of drug targeting. Nanomaterial-based drug systems provide the advantage of being able to penetrate cell membranes through minuscule capillaries in the cell wall of rapidly dividing tumor cells, while at the same time having low cytotoxicity toward normal cells. Nanomaterials have been found to have favorable interaction with the brain blood vessel endothelial cells of mice, and thus they might have the possibility of being transported to other brain tissues, making them potential neutron capture therapy agents.^11^, ^12^ In theory, in BNCT and GdNCT nanomaterials, a large number of boron and gadolinium atoms could be incorporated, thereby lowering the dose requirement for delivering critical amounts of ^10^B and Gd to tumor cells. Accordingly, improvement of the drug storage capacity is very important.^7^

Magnetic nanoparticles are being studied in terms of their highly promising applications in biology and medicine, including magnetic cell separation, magnetic resonance imaging (MRI) contrast enhancement, and magnetic targeted drug delivery for cancer magnetic hyperthermia.^10^ MRI is a noninvasive technique for obtaining real-time three-dimensional images of the interior of solids (particularly cells), tissues, and organs. But magnetic nanoparticles tend to aggregate due to strong magnetic dipole–dipole attraction between particles brought together by van der Waals interparticle attractions and their inherently large surface energy. Therefore, coating agents, such as surfactants or capping ligands with some specific functional groups, have been used to modify these particles in order to prevent the sedimentation and to obtain better surface properties.^13^

Silicates have attracted significant interest because of their rich structural chemistry, which makes the development of new structures and functionalities possible. Amorphous silica with a nontoxic nature, tunable diameter, and very high specific surface area with abundant Si–OH bonds on the surface are promising candidates for use as carriers in drug delivery systems. Thus, nanocomposites of SiO_2_ and magnetic particles have attracted considerable attention in targeted drug delivery because of the high surface area and magnetic separability.^14^

Crystalline FeBO_3_ material is known for its unique magnetic and acoustic resonance properties.^15^ In contrast, GdFeO_3_ shows promising relaxivity properties and has potential as an MRI contrast agent.^16^ Fe_3_O_4_ has been considered to be an ideal candidate for biological applications due to its special magnetic properties, lack of toxicity, and good biocompatibility.^17^ The nanocomposites of these materials can carry an active agent (drug) and be guided to the target site inside the body, facilitating therapeutic efficiency and minimizing damage to normal tissue due to drug toxicity.

In recent years, several different routes have been used to synthesize biofunctional magnetic nanocomposites.^18^ The gel combustion method has been developed and widely used to prepare phase-pure nanopowders.^19^ The method has the advantages of using inexpensive precursors, requiring a simple experimental process, and resulting in an ultrafine, homogenous powder. Chavan and Tyagi used a combustion method to produce GdFeO_3_ nanoparticles with sizes in the range of 40–65 nm.^20^ However, there have been only a few reports on combinations of magnetic Fe_3_O_4_ with FeBO_3_ and GdFeO_3_ nanoparticles. For this reason, we developed a route consisting of encapsulating preformed ^10^B, Gd and Fe_3_O_4_ nanoparticles into silica. The aim was to obtain core shell nanoparticles, denoted Fe^10^BO_3_/Fe_3_O_4_/SiO_2_, and GdBO_3_/Fe_3_O_4_/SiO_2_, which will 1) improve the drug storage capacity, 2) have sufficiently powerful magnetic properties, 3) form a stable dispersion at physiological pH, and 4) have facile surface chemistry to allow the use of coupling agents, such as commercially available alkoxysilane derivatives.

The reactions described herein were generally performed under air or argon, and Fe^10^BO_3_/Fe_3_O_4_/SiO_2_ and GdFeO_3_/Fe_3_O_4_/SiO_2_ nanocomposites were prepared via the route shown in Scheme 1. Powder X-ray diffraction (XRD) was used to investigate the variations in structure of the samples produced by the gel combustion method under different conditions (Figures 1). Fe^10^BO_3_ and GdFeO_3_ crystallized as a phase-pure material at calcination temperatures as low as 680 °C in 2 h (Figure 1 a,c). The diffraction peaks of the product can be readily indexed to the pure Fe^10^BO_3_ and GdFeO_3_ (Joint Committee on Powder Diffraction Standard (JCPDS) card no. 76-0701 and 78-0451, respectively). No additional peaks for other phases or impurities were found. These results demonstrate that well-crystallized Fe^10^BO_3_ and GdFeO_3_ can be obtained using the gel combustion technique. After coating with Fe_3_O_4_, the product is a nanocomposite of Fe^10^BO_3_ or GdFeO_3_ and Fe_3_O_4_ (Figure 1 b,d), suggesting that a hybrid material, composed of Fe^10^BO_3_ or GdFeO_3_ and Fe_3_O_4_, had formed. No peaks of other phases were detected, indicating that no other reaction occurred between the core and the shell during the synthesis.

**Scheme 1:**
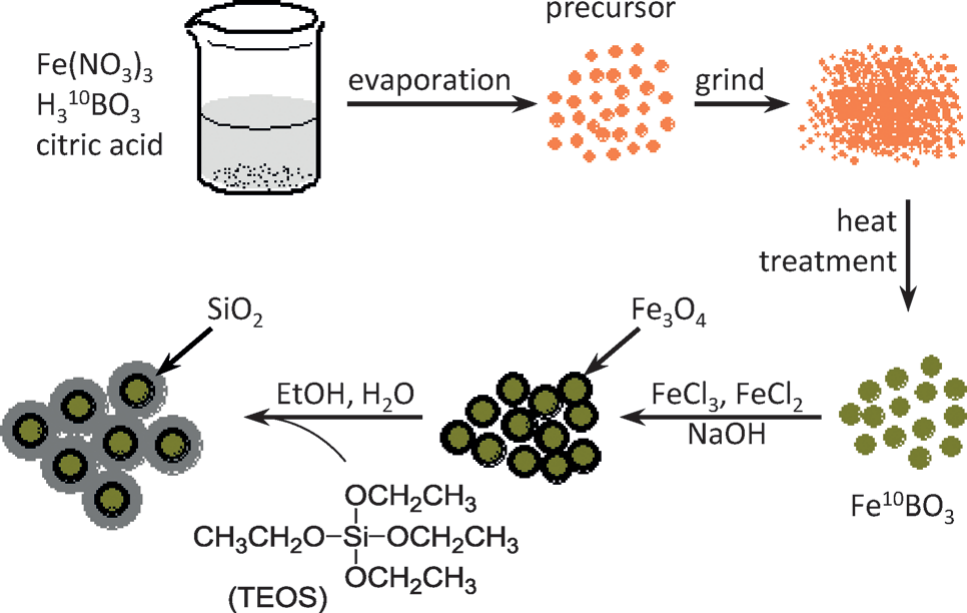
Schematic representation of the fabrication of Fe^10^BO_3_/Fe_3_O_4_/SiO_2_ nanocomposites. TEOS=tetraethoxysilane.

**Figure 1:**
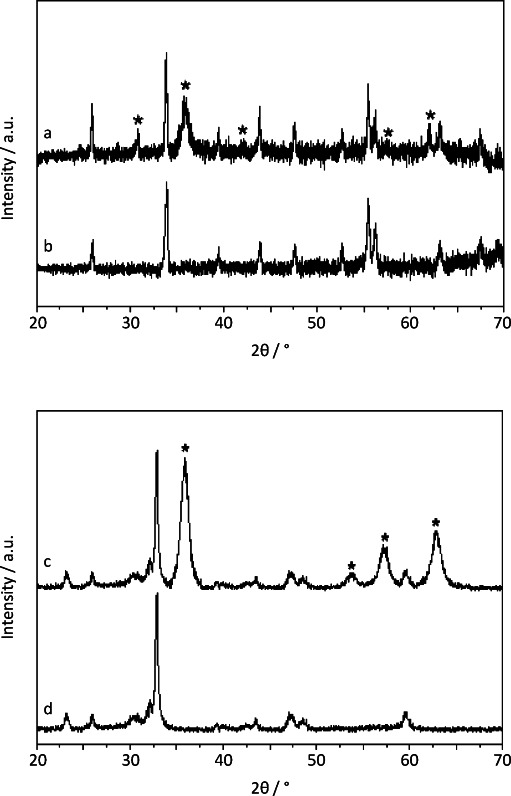
Powder X-ray diffraction (XRD) patterns of the Fe^10^BO_3_ (top) and GdFeO_3_ (bottom) sample before (a and c) and after (b and d) Fe_3_O_4_ and SiO_2_ coating. * Characteristic diffraction peaks of Fe_3_O_4_.

The typical microstructure of the sample was examined by transmission electron microscopy (TEM) analysis. Figure 2 shows the TEM images of the samples before and after coated with Fe_3_O_4_ and SiO_2_. For pure Fe^10^BO_3_ and GdFeO_3_, the TEM images indicate that the nanoparticles are spherical and the particle diameter is about 60 nm (Figure 2 a,d). When coated with Fe_3_O_4_, the particles tend to aggregate due to strong magnetic dipole–dipole attractions between them (Figure 2 b,e). After the particles were coated with SiO_2_, there was a thin layer of amorphous silica covering the surface (Figure 2 c,f).

**Figure 2:**
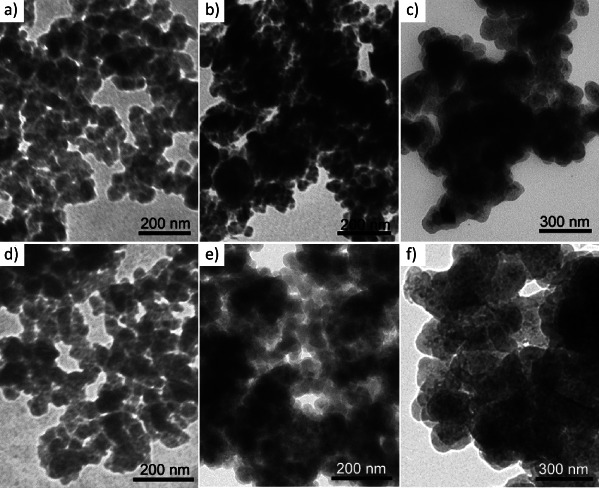
Transmission electron microscopy (TEM) images of the samples after each coating step. a) Fe^10^BO_3_, b) Fe^10^BO_3_/Fe_3_O_4_, c) Fe^10^BO_3_/Fe_3_O_4_/SiO_2_, d) GdFeO_3_, e) GdFeO_3_/Fe_3_O_4_, f) GdFeO_3_/Fe_3_O_4_/SiO_2_.

In order to deduce the composition of the nanocomposites, energy-dispersive X-ray spectroscopy (EDS) analysis was carried out (Figure 3). Before coating with Fe_3_O_4_ and SiO_2_, the EDS specta of the samples depict no other peaks except those for Fe^10^BO_3_ (Fe and O, Figure 3 a) and GdFeO_3_ (Gd, Fe and O, Figure 3 c), indicating the high purity of the composites obtained by the method described above. After coating with Fe_3_O_4_ and SiO_2_, the EDS spectra indicate that Fe, Si and O composited for Fe^10^BO_3_/Fe_3_O_4_/SiO_2_ and Gd, Fe, Si and O for GdFeO_3_/Fe_3_O_4_/SiO_2_ nanocomposites. Because boron is a light element, EDS cannot detect its presence, but the pattern seen in the XRD spectrum indicates that the compound is a composite of Fe^10^BO_3_ and Fe_3_O_4_.

**Figure 3:**
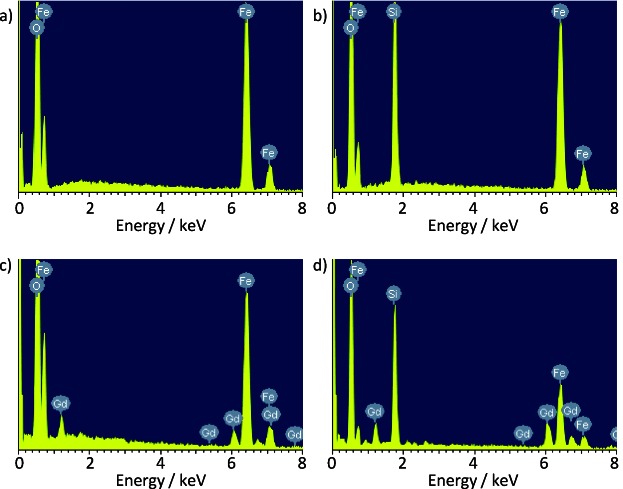
Energy-dispersive X-ray spectroscopy (EDS) of samples before and after Fe_3_O_4_ and SiO_2_ coatings. a) Fe^10^BO_3_, b) GdFeO_3_, c) Fe^10^BO_3_/Fe_3_O_4_/SiO_2_, d) GdFeO_3_/Fe_3_O_4_/SiO_2_.

Fourier-transformed infrared (FT-IR) spectroscopy was used to identify the surface functional groups of the samples. Figure 4 shows the FT-IR spectra of Fe^10^BO_3_/Fe_3_O_4_/SiO_2_ and GdFeO_3_/Fe_3_O_4_/SiO_2_ nanocomposites in the region of 500 cm^−1^ to 4000 cm^−1^. A broad band with a maximum at 3437.8 cm^−1^ is attributed to the O–H stretching vibrations in both the Si–OH groups and some physisorbed water, which is confirmed by the presence of an H_2_O deformation band (bending vibration of H–O–H) at 1633.6 cm^−1^. For the Fe^10^BO_3_/Fe_3_O_4_/SiO_2_ sample, bands at 1254.8 and 1965.6 cm^−1^ are due to vibrations of the B–O bond and other bonds attached to the B or the O of the B–O bond.^21^–^23^ The band at ∼1050 cm^−1^ corresponds to υ(Si–OH); the bands at ∼1100 and ∼860 cm^−1^ correspond to υ_asym_(Si–O–Si) and υ_sym_(Si–O–Si) modes, respectively. The absorption at 767.7 cm^−1^ could be attributed to the O–H stretching vibration on the surface of Fe_3_O_4_. An additional absorption at 667 cm^−1^ could be attributed to the Fe–O–B bending vibration analogous to Si–O–B.^23^,^24^ A very small band shoulder at ∼560–580 cm^−1^, observed in the IR spectra, can be assigned to υ(Fe–O) and υ(Gd–O) in Fe–O–Fe and Gd–O–Fe systems, respectively.^25^–^27^ These results indicate that Fe_3_O_4_ and SiO_2_ are immobilized on the surfaces of the Fe^10^BO_3_ and GdFeO_3_ nanoparticles.

**Figure 4:**
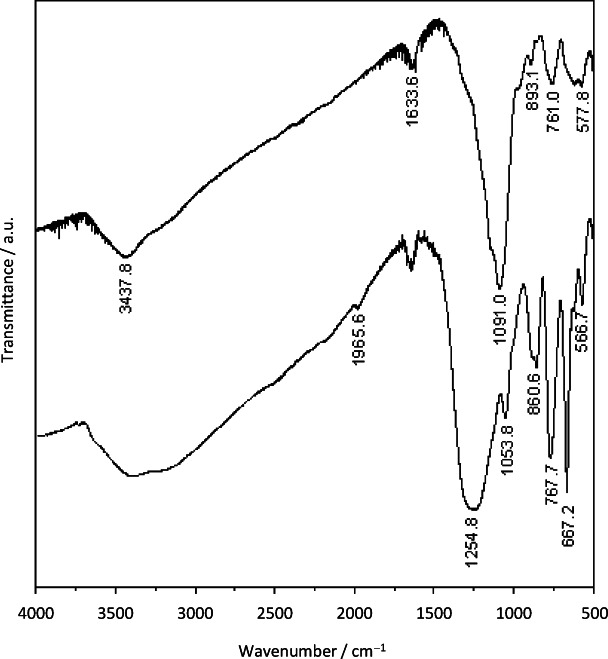
Fourier-transformed infrared (FT-IR) spectra of nanocomposites a) Fe^10^BO_3_/Fe_3_O_4_/SiO_2_ and b) GdFeO_3_/Fe_3_O_4_/SiO_2_.

The magnetization curve measured at room temperature for the Fe^10^BO_3_/Fe_3_O_4_/SiO_2_ and GdFeO_3_/Fe_3_O_4_/SiO_2_ nanocomposites shows a small hysteresis loop suggesting that the nanocomposites have ferromagnetic behavior (Figure 5). It has been reported that magnetic Fe_3_O_4_ particles exhibit super-paramagnetic behavior when the particle size decreases to below a critical value, generally around 20 nm.^28^ The Fe_3_O_4_ particles are aggregated and connected to form larger particles, resulting from the ferromagnetic behavior. The magnetization saturation values for Fe^10^BO_3_/Fe_3_O_4_/SiO_2_ and GdFeO_3_/Fe_3_O_4_/SiO_2_ are about 22.6 and 48.7 emu g^−1^, respectively. These values are lower than that of pure Fe_3_O_4_ (87 emu g^−1^), probably because of the small percentage of Fe_3_O_4_ in the nanocomposites. The magnetic separation ability of the sample was tested in water by placing a magnet near the glass bottle containing a suspension of the nanocomposite. The deep brown and black particles were attracted towards the magnet (Figure 5, inset). This property will provide an easy and efficient way to separate the Fe^10^BO_3_/Fe_3_O_4_/SiO_2_ and GdFeO_3_/Fe_3_O_4_/SiO_2_ nanocomposites from a suspension system and to carry drugs to targeted locations under an external magnetic field. These results indicate that the nanocomposites possess excellent magnetic responsiveness. The magnetic property permits the use of the biofunctional nanoparticles in biomedical applications because they have sufficiently strong magnetization for efficient magnetic separation in the presence of an externally applied magnetic field.^29^

**Figure 5:**
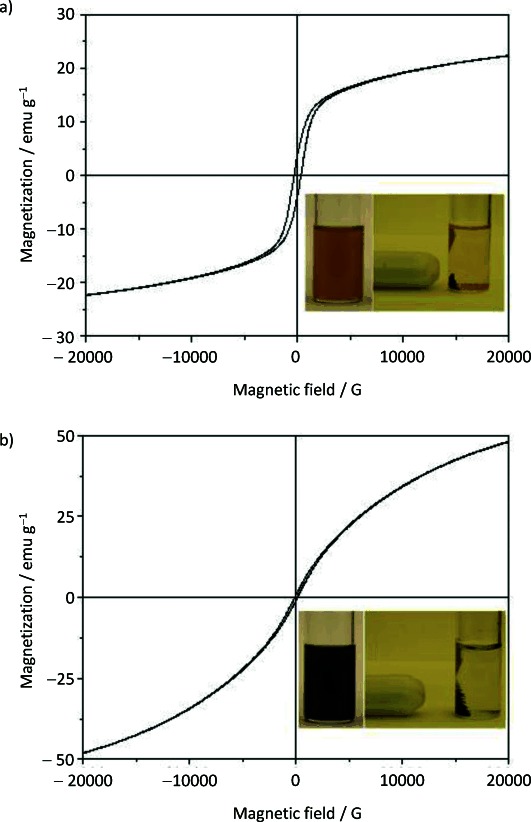
Measured magnetic hysteresis loops for nanocomposites a) Fe^10^BO_3_/Fe_3_O_4_/SiO_2_ and b) GdFeO_3_/Fe_3_O_4_/SiO_2_. Inset: photograph of magnetic targeting under an external magnet.

A novel kind of magnetic sphere with ^10^B or Gd and Fe_3_O_4_ nanoparticles encapsulated in the cores of silica shells has been fabricated. The nanocomposite spheres, which combine the advantages of silica and magnetic carrier technology, are likely to be applied in targeted drug delivery. The main focus of this research was to synthesize novel neutron capture therapy materials that are both effective and relatively harmless to the patient. The next stage of this research involves the biological evaluation of the two nanocomposites reported here and is currently underway in our laboratories.

## Experimental Section

The chemicals used in this study, such as Gd(NO_3_)_3_⋅6H_2_O, Fe(NO_3_)_3_⋅9H_2_O, FeCl_2_⋅4H_2_O, FeCl_3_, H_3_^10^BO_3_, citric acid, tetraethoxysilane (TEOS), were all of analytical reagent grade, purchased from Aldrich, and used as received without any further purification. Water used was deionized and doubly distilled.

The combustion synthesis utilized Fe(NO_3_)_3_⋅9H_2_O (1.92 g, 0.0048 mol) and H_3_^10^BO_3_ (0.92 g, 0.015 mol) as the starting materials. Citric acid was used as the fuel, and chelation was in the ratio 1:1.25. The precursors and fuel were mixed in water (50 mL) to obtain a transparent aqueous solution, which on thermal dehydration resulted in a highly viscous liquid. On further heating to high temperature (190 °C), the viscous liquid swelled and dried. The residue was then ground to obtain a powder, which was then subjected to further heat treatment at 680 °C for 2 h under an argon atmosphere to isolate Fe^10^BO_3_ nanoparticles. In the synthesis of GdFeO_3_ nanoparticles, the H_3_^10^BO_3_ was replaced by Gd(NO_3_)_3_⋅6H_2_O.

The obtained Fe^10^BO_3_ or GdFeO_3_ nanoparticles (0.20 g) were ultrasonically dispersed in water (50 mL), then FeCl_3_ (0.81 g, 0.005 mol) and FeCl_2_ (0.5 g, 0.0025 mol) were added, followed by the dropwise addition of aq NaOH (0.37 g in 30 mL H_2_O) with vigorous stirring. The resulting black suspension was further stirred for 30 min at RT to form black magnetite nanocomposites, which were first separated magnetically, washed several times with deionized water and EtOH, and then vacuum dried at 60 °C for 5 h. All main synthetic steps using Fe_3_O_4_ were carried out by passing argon through the solution to avoid possible oxygen contamination during the operations.

Silica-coated Fe^10^BO_3_/Fe_3_O_4_ and GdFeO_3_/Fe_3_O_4_ nanocomposites were produced by hydrolysis of TEOS on the surfaces of these magnetic nanocomposites. The freshly prepared Fe^10^BO_3_/Fe_3_O_4_ or GdFeO_3_/Fe_3_O_4_ nanocomposite powder (0.40 g) was ultrasonically redispersed in a solution containing EtOH (120 mL) and water (14 mL). The solution was then loaded into a three-necked bottle, and the pH of the solution was adjusted to 9 with NH_4_OH (3.0 mL, 14.8 m), and TEOS (2 mL, 0.009 mol) was added to the mixture under vigorous stirring. After 24 h, the particles were separated magnetically, washed with deoxygenated distilled water and anhydrous EtOH, and then vacuum dried at 50 °C overnight to collect the silica-coated nanocomposites Fe^10^BO_3_/Fe_3_O_4_/SiO_2_ or GdFeO_3_/Fe_3_O_4_/SiO_2_.

The phases of the final products were identified using an X-ray diffractometer (Rigaku D/max-2500 VPC) with Ni-filtered Cu Kα radiation at a scanning rate of 0.02° s^−1^ from 20° to 80°. A Hitachi model H800 transmission electron microscope was used for determining the size and shape of the powder particles. Fourier-transform infrared (FT-IR) spectra were recorded using a MAGNA 550 FT-IR spectrometer on samples embedded in KBr pellets. Magnetization measurements were performed using an ACBH-100K B-H hysteresis loops measuring instrument. All measurements were performed at room temperature.
